# Tumor mutational burden assessed by targeted NGS predicts clinical benefit from immune checkpoint inhibitors in non‐small cell lung cancer

**DOI:** 10.1002/path.5344

**Published:** 2019-10-24

**Authors:** Ilaria Alborelli, Katharina Leonards, Sacha I Rothschild, Laura P Leuenberger, Spasenija Savic Prince, Kirsten D Mertz, Severin Poechtrager, Martin Buess, Alfred Zippelius, Heinz Läubli, Jasmin Haegele, Markus Tolnay, Lukas Bubendorf, Luca Quagliata, Philip Jermann

**Affiliations:** ^1^ Department of Medical Genetics and Pathology University Hospital Basel Basel Switzerland; ^2^ Laboratory of Cancer Immunology, Department of Biomedicine University Hospital Basel Basel Switzerland; ^3^ Department of Medical Oncology, Department of Internal Medicine University Hospital Basel Basel Switzerland; ^4^ Department of Pathology Cantonal Hospital Baselland Liestal Switzerland; ^5^ Department of Medical Oncology St. Claraspital Basel Switzerland

**Keywords:** TMB, NSCLC, cancer immunotherapy, checkpoint inhibitor, NGS

## Abstract

In non‐small cell lung cancer (NSCLC), immune checkpoint inhibitors (ICIs) significantly improve overall survival (OS). Tumor mutational burden (TMB) has emerged as a predictive biomarker for patients treated with ICIs. Here, we evaluated the predictive power of TMB measured by the Oncomine™ Tumor Mutational Load targeted sequencing assay in 76 NSCLC patients treated with ICIs. TMB was assessed retrospectively in 76 NSCLC patients receiving ICI therapy. Clinical data (RECIST 1.1) were collected and patients were classified as having either durable clinical benefit (DCB) or no durable benefit (NDB). Additionally, genetic alterations and PD‐L1 expression were assessed and compared with TMB and response rate. TMB was significantly higher in patients with DCB than in patients with NDB (median TMB = 8.5 versus 6.0 mutations/Mb, Mann–Whitney *p* = 0.0244). 64% of patients with high TMB (cut‐off = third tertile, TMB ≥ 9) were responders (DCB) compared to 33% and 29% of patients with intermediate and low TMB, respectively (cut‐off = second and first tertile, TMB = 5–9 and TMB ≤ 4, respectively). TMB‐high patients showed significantly longer progression‐free survival (PFS) and OS (log‐rank test *p* = 0.0014 for PFS and 0.0197 for OS). While identifying different subgroups of patients, combining PD‐L1 expression and TMB increased the predictive power (from AUC 0.63 to AUC 0.65). Our results show that the TML panel is an effective tool to stratify patients for ICI treatment. A combination of biomarkers might maximize the predictive precision for patient stratification. Our study supports TMB evaluation through targeted NGS in NSCLC patient samples as a tool to predict response to ICI therapy. We offer recommendations for a reliable and cost‐effective assessment of TMB in a routine diagnostic setting. © 2019 The Authors. *The Journal of Pathology* published by John Wiley & Sons Ltd on behalf of Pathological Society of Great Britain and Ireland.

## Introduction

The outcome of metastatic non‐small cell lung cancer (NSCLC) patients has been considerably improved by the use of immune checkpoint inhibitors (ICIs) targeting programmed cell death receptor‐1 (PD‐1) or its ligand (PD‐L1) 1, 2, 3. Because only a subset of patients respond to ICI therapy 4, 5, it is critical to identify biomarkers that can predict treatment outcome. PD‐L1 expression in tumor and/or tumor‐associated immune cells is an established biomarker to predict benefit from PD‐1/PD‐L1 checkpoint inhibitor therapy 6, 7. However, since PD‐L1 expression has limited predictive power 3, 7, 8, 9, new biomarkers are needed to improve the precision of clinical decisions and identify potential responders to ICI therapy. The presence of tumor‐specific neoantigens is associated with increased immunogenicity 10, leading to the hypothesis that tumors presenting a higher number of neoantigens may respond better to immunotherapy 11, 12, 13. Recently, tumor mutational burden (TMB), an indirect measure of tumor‐derived neoantigens, has emerged as a promising biomarker for ICI patient stratification. The clinical utility of TMB in ICI treatment of NSCLC has been supported by few seminal studies in which the mutational load was measured through whole‐exome sequencing (WES) 14, 15, 16 or targeted sequencing 17, 18. In all of these studies, high mutational load correlated with increased response rate to ICIs and longer progression‐free survival (PFS) in NSCLC patients. No correlation was observed between PD‐L1 expression and mutational load, suggesting that these biomarkers characterize different patient populations.

Given these promising results, much effort is currently being put into establishing TMB analysis in routine diagnostic laboratories. Technical limitations as well as cost, turnaround time (TaT), and tissue availability render targeted panel sequencing more suitable for clinical use than WES. Additionally, several pre‐analytical and analytical variables influence the accuracy of TMB assessment, calling for standardization and harmonization initiatives. The technical performance of several commercially available panels has been matched to WES results, showing an excellent correlation 17, 19, 20, 21. Nevertheless, clinical samples present with a number of challenges, as tissue biopsies are heterogeneous in tumor cellularity, cell viability, quality, and yield of the extracted DNA. Another pre‐analytical factor particularly relevant when working with formalin‐fixed, paraffin‐embedded (FFPE) samples is the presence of deamination artifacts caused by formalin fixation. Taken together, these and other factors have contributed to a significant reduction in the number of patients for whom TMB could be assessed in several clinical trials (59% evaluable patients in CheckMate 26 14, 58% in CheckMate 227 18 and 34% in CheckMate 568).

Our study is the first to evaluate the clinical validity of the Oncomine™ Tumor Mutational Load (TML – Thermo Fisher Scientific) assay in FFPE samples collected from a cohort of 76 NSCLC patients treated with ICI therapy. We show that the Oncomine™ TML assay can be used to stratify patients according to their likelihood to respond to ICI therapy, supporting the application of this panel in routine diagnostics and clinical studies. We also offer recommendations on how to approach common pre‐analytical and analytical challenges when measuring TMB. Overall, our results demonstrate that using TMB as a biomarker may lead to higher accuracy in predicting response to ICI agents.

## Materials and methods

### Patient cohort

Seventy‐six NSCLC patients treated with ICIs between April 2013 and January 2018 at the University Hospital Basel; the Cantonal Hospital Baselland, Switzerland; and the St Clara Hospital Basel were selected for this study. Eligible patients were defined as having a histologically confirmed diagnosis of NSCLC, sufficient tissue material to perform TMB analysis, and PFS and overall survival (OS ≥1 month) data. Additionally, we collected data on treatment history, smoking status, PD‐L1 expression, and tumor stage. Patients were characterized as having either durable clinical benefit (DCB) or no durable benefit (NDB) in addition to complete/partial response (CR/PR), stable disease (SD) or progressive disease (PD) as best response (RECIST 1.1). DCB was defined as CR/PR or SD for at least 6 months, whereas NDB was defined as progression within 6 months from start of ICI treatment. Baseline was defined as the start of ICI treatment.

### Tumor samples

Paraffin‐embedded tumor biopsies were collected from 76 advanced NSCLC patients. The study was approved by the local Ethical Review Board (Ethikkommission Nordwestschweiz, Project‐ID 2018–01751) and performed in compliance with all relevant ethical regulations. Tissue biopsies were obtained at the time of first diagnosis, except for *n* = 6 patients for whom biopsy was available at later disease stages. Tumor cell content was assessed through examination of hematoxylin and eosin‐stained slides by at least two thoracic pathologists (LB, SSP, KM).

### PD‐L1 immunohistochemistry

PD‐L1 expression was assessed using the Ventana SP263 assay on the BenchMark platform (Ref 740–4907; Ventana, Tucson, AZ, USA). Two experienced surgical pathologists (SSP and KM) evaluated the staining for PD‐L1 tumor proportion score (TPS), which represents the percentage of PD‐L1‐positive tumor cells (TCs) relative to all TCs present in the sample. PD‐L1 cell positivity was defined as partial or complete membrane staining, irrespective of staining intensity. A minimum of 100 viable tumor cells were required for evaluation of PD‐L1 expression. PD‐L1 scoring was available for 67 of the 76 patients.

### DNA extraction and NGS library preparation

For DNA extraction, four or five FFPE tissue sections of 10 μm thickness were cut and deparaffinized using xylol. DNA extraction from tissue was performed using the column‐based RecoverAll™ Extraction Kit (Thermo Fisher Scientific, Waltham, MA, USA) according to the manufacturer's instructions. TMB was assessed using a 409‐gene targeted NGS assay that detects variants in all coding regions (Oncomine™ TML Assay, Thermo Fisher Scientific). For NGS library preparation, 5–40 ng of DNA was used, depending on the availability of input material. If DNA input was less than 20 ng, an additional PCR cycle was added during target enrichment. The libraries were purified using Agencourt AmpureXP beads (Beckman Coulter, Indianapolis, IN, USA) and quantified by qPCR using the Ion Universal Quantitation Kit (Thermo Fisher Scientific). For samples showing more than five deamination artifacts after sequencing, library preparation was repeated pretreating DNA with UDG (uracil‐DNA glycosylase, Thermo Fisher Scientific).

### Sequencing

Sequencing runs were planned on the Torrent Suite Software™ v5.8 and libraries were diluted to 50 pm, combined in batches of five libraries, loaded on an Ion 540™ chip using the Ion Chef™ instrument, and sequenced on an Ion S5XL™ instrument (Thermo Fisher Scientific). Raw data were processed automatically on the Torrent Server™ and aligned to the hg19 reference genome. An average of 18 500 000 (9 000 000–28 000 000) reads were obtained per sample, with 98% (69.6–99.6%) on‐target reads, 92.5% (65.2–97.5%) read uniformity, and 1170X (500X–1800X) average coverage. Sequencing data were then uploaded in BAM format to the Ion Reporter™ Analysis Server for TML score calculation and variant calling.

### Sequencing data analysis and TMB calculation

Variant detection and TMB calculation were performed on Ion Reporter™ Analysis Software v5.10 (IR) using the Oncomine™ Tumor Mutation Load w2.0 workflow. The default limit of detection (LOD) was set at 5% allelic frequency (AF) and adjusted to 10%, depending on the presence of potential deamination artifacts. Germline variants were filtered automatically by cross‐referencing with UCSC common SNPs, ExAC, 10 000 Genomes, and 5000Exomes databases. Somatic variants in homopolymer stretches longer than 4 bp were also excluded. TMB was calculated by dividing the number of somatic missense and nonsense mutations and coding indels by the number of exonic bases with at least 60X coverage and expressed as the number of mutations per megabase. TMB values were rounded to whole numbers in order to account for the technical variability of the assay. Potential deamination artifacts were defined as C:G>T:A mutations with an allelic frequency less than 15% in coding regions. We considered only samples with < 3 artifacts for tissue specimens with tumor cell content (TCC) < 50%, and ≤ 1 for samples with TCC ≥ 50%. For samples exceeding the allowed number of artifacts, data were re‐analyzed using an AF of 10%. If estimated artifacts were still greater than 3, samples were excluded from the study. Data for concordance analysis were available for 47 out of 76 samples of our cohort. Samples were initially analyzed in routine diagnostic with an orthogonal method, in particular Sanger Sequencing (*n* = 19) or targeted NGS panels using the default analysis pipeline in IR (Oncomine™ Solid Tumor *n* = 14, Oncomine™ Focus *n* = 7, Cancer Hotspot v2 *n* = 6, Oncomine™ Comprehensive v3 *n* = 1). For the analysis of specific gene alterations, both germline and somatic mutations were considered.

### Statistical data analysis

To assess significance for baseline clinical characteristics, an unpaired *t*‐test was used for age and tumor cell content, and Fisher's exact test for sex, tumor histology, tumor type, smoking status, PD‐L1 expression, immunotherapy, number of lines, stage, and durable clinical benefit (Table 1). For correlation between TMB and DCB rate, the Mann–Whitney test was used. For multiple comparisons, the Kruskal–Wallis test including Dunn's multiple comparisons test was applied. For correlations, Spearman's rank coefficient was used. Survival curves were analyzed using a log‐rank test. *P* values were two‐sided and considered significant if less than 0.05. Statistical analyses were performed using GraphPad Prism version 8 (GraphPad Software Inc, San Diego, CA, USA) and R software package (https://www.r-project.org) version 3.4 or later.

**Table 1:** Baseline characteristics of NSCLC patients assessed for tumor mutational burden

Patient characteristics	All patients (*n* = 76) No (%)	TMB low and int (*n* = 51) No (%)	TMB high (*n* = 25) No (%)	*P* value
Age (years)				0.907
Median (range)	66 (31–90)	65 (49–79)	67 (31–90)	
Sex (*N*)				0.615
Male	47 (62)	30 (59)	16 (68)	
Female	29 (38)	21 (41)	8 (32)	
Tumor histology at diagnosis (*N*)				>0.999
Adenocarcinoma	70 (92)	47 (92)	23 (92)	
Squamous cell carcinoma	6 (8)	4 (8)	2 (8)	
Tumor type (*N*)				0.043
Primary tumor	47 (62)	36 (71)	11 (44)	
Metastasis/lymph node	29 (38)	15 (29)	14 (56)	
Tumor cell content (%)				0.213
Median (range)	60 (20–95)	60 (20–95)	60 (20–90)	
Immunotherapy (*N*)				>0.999
Nivolumab	60 (79)	40 (78)	20 (80)	
Pembrolizumab	10 (13)	9 (18)	1 (4)	
Atezolizumab	3 (4)	2 (4)	1 (4)	
Other (Nivolumab + ipilimumab)	3 (4)	0 (0)	3 (12)	
No of lines before I‐O (*N*)				0.724
First (0)	11 (14)	7 (14)	4 (16)	
Second (1)	39 (51)	30 (59)	9 (36)	
Third (2)	10 (13)	6 (12)	4 (16)	
Fourth (3)	2 (3)	0 (0)	2 (8)	
Not available	13 (17)	8 (16)	5 (20)	
Smoking status (*N*)				0.155
Never	10 (13)	9 (18)	1 (4)	
Current/former	60 (79)	39 (76)	21 (84)	
Not available	6 (8)	3 (6)	3 (12)	
PD‐L1 (*N*)				>0.999
< 1%	28 (37)	19 (37)	9 (36)	
≥ 1%	39 (51)	27 (53)	12 (48)	
Not available	9 (12)	5 (10)	4 (16)	
Stage at diagnosis (*N*)				>0.999
I–III	25 (33)	17 (33)	8 (32)	
IV	49 (64)	32 (63)	17 (68)	
Durable clinical benefit (*N*)				0.013
DCB	32 (42)	16 (31)	16 (64)	
No DCB	44 (58)	35 (69)	9 (36)	

## Results

### Pre‐analytical and analytical variables influence the TMB value

To address common challenges encountered when handling clinical samples and offer a recommendation for accurate TMB analysis, we investigated the impact of pre‐analytical and analytical variables on TMB estimation in our patient cohort. Several factors influence TMB robustness. Importantly, targeted panel size has been proven to strongly affect the precision of TMB estimation 22. The panel used in this study has been technically validated 20, 21, confirming its ability to detect somatic mutations with a strong correlation (*r* ^2^ = 0.986) to WES across several tumor types. Based on the technical variability of TMB measurement with this assay, we decided to express all TMB values in this study as rounded whole numbers. Our samples showed a high degree of heterogeneity, with TCC ranging from 20% to 95% and the storage period of FFPE blocks ranging from 0 to 5 years. Prolonged storage of FFPE blocks has been shown to influence DNA quality and the frequency of deamination artifacts 23, 24.

**Figure 1:**
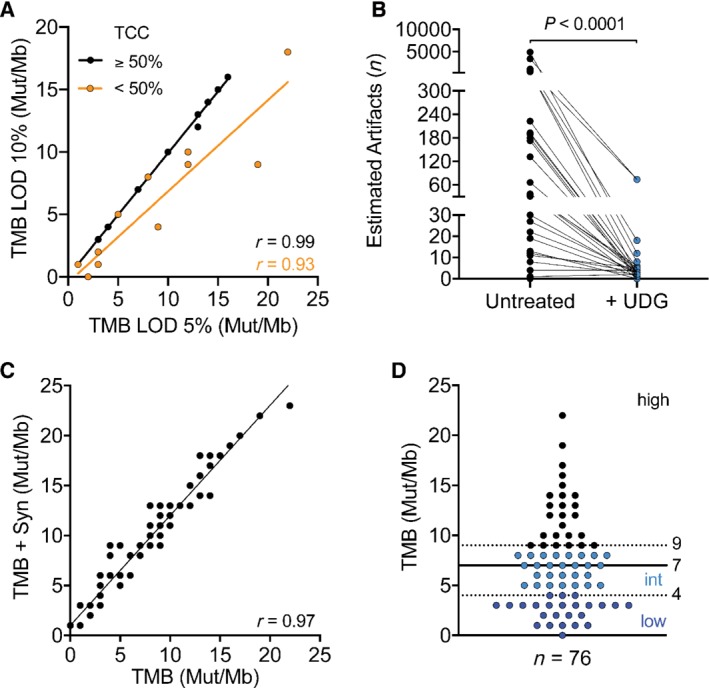
Evaluation of pre‐analytical factors affecting TMB measurement. (A) Comparison of TMB analysis workflows based on either 5% or 10% limit of variant detection (LOD). Linear regression between the two workflows was calculated for samples with either ≥ 50% tumor cell content (TCC) (Spearman *r* = 0.99) or < 50% (orange; Spearman *r* = 0.93). (B) Estimation of deamination artifacts before and after treatment (blue dots) of input DNA with uracil‐DNA glycosylase (UDG) (Mann–Whitney *p* < 0.0001). (C) Linear regression of TMB assessed by using only non‐synonymous versus all mutations (Spearman *r* = 0.97). (D) Distribution of TMB values across the sample cohort (*n* = 76). Dashed lines indicate tertiles used to define low (dark blue dots, ≤ 4 Mut/Mb), intermediate (light blue dots, 5 < *x* < 9 Mut/Mb), and high TMB (≥ 9 Mut/Mb). Solid line indicates the median of all samples (7 Mut/Mb).

Both TCC and the presence of FFPE artifacts affect the sensitivity and specificity of TMB assessment, influencing allelic frequency and mutational count, respectively. Thus, these factors should be considered particularly when choosing the limit of variant detection (LOD) used for TMB calculation, which has been set at 5% or 10% of AF in previous studies 15, 21, 25 and reviewed in ref 26. We addressed this issue by analyzing the correlation of LOD 5% and 10% in samples with high TCC (≥ 50%) or low TCC (< 50%) (Figure 1A). As expected, the difference between TMB calculated with 5% and 10% LOD workflow was more pronounced in samples with estimated TCC < 50%, indicating that an LOD of 5% improves the accuracy of TMB measurement in samples with low TCC (Figure 1A).

Next, we examined the effect of cytosine deamination resulting from formalin fixation, a phenomenon common to FFPE specimens and known to lead to false‐positive variant calls 24, 27. The Ion Reporter™ TMB analysis workflow estimates the number of deamination artifacts by classifying each C:G>T:A variant with an AF < 15% as a potential artifact. We detected mutation signatures consistent with the presence of deamination artifacts in 37% of the samples, with the number of estimated artifacts ranging from 4 to 4931 (Figure 1B). As the presence of artifacts could potentially lead to significant overestimation of TMB, we treated DNA from these samples with uracil‐DNA glycosylase (UDG), an enzyme that selectively digests uracil‐containing nucleic acids, reducing sequencing artifacts 28, 29. Treatment with UDG led to a 92% reduction of estimated deamination artifacts, with 65% of treated samples reverting to less than five estimated artifacts (Figure 1B). Lastly, the type of variants considered for TMB calculation has been discussed in several reports (reviewed in 30, 31). In particular, the inclusion of synonymous mutations has been debated and found to be potentially useful when using smaller sized panels for TMB assessment 20, 22. The default Ion Reporter™ TML analysis workflow considers only non‐synonymous variants for TMB calculation, but the initial variant caller also detects synonymous mutations. To assess the impact of synonymous mutations, we calculated the TMB value for each sample considering also synonymous variants and compared it with the TMB values obtained by counting non‐synonymous variants only. Our data show a strong correlation between the two sets of TMB values (Spearman *r* = 0.97) (Figure 1C and supplementary material, Figure S1A), indicating that both analysis pipelines yield similar relative results. For the purpose of this study, all TMB calculations were performed using the default workflow provided by the manufacturer, considering only non‐synonymous mutations.

Taking together all of the above factors, our final dataset comprises the TMB values of 76 patient samples withstanding our quality control assessment (Figure 1D). Median TMB was 7 mutations (Mut)/Mb and TMB values ranged from 0 to 22. Previous studies have categorized TMB into low, intermediate, and high mutational burden 14, 32. Similarly, we categorized our dataset according to tertiles of low (≤ 4 Mut/Mb), intermediate (5 < *x* < 9 Mut/Mb), and high TMB (≥ 9 Mut/Mb).

### TMB correlates with clinical benefit

The baseline characteristics of 76 ICI‐treated NSCLC patients are described in Table 1. Median age was 66 years (range 31–90 years); 62% of the patients were male; 79% were current or former smokers; and 92% had adenocarcinoma histology. Seventy‐nine percent of our cohort received nivolumab monotherapy, and the majority of patients were treated in the second‐line setting (51%).

**Figure 2:**
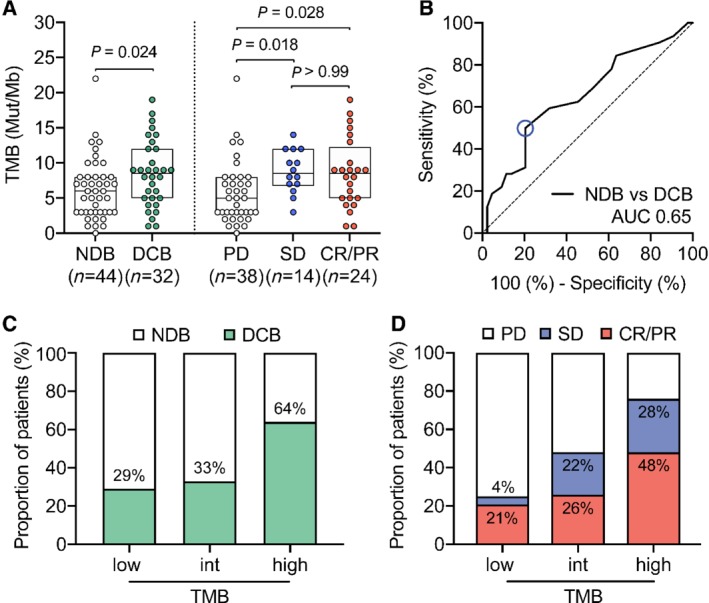
TMB correlates with response to ICI treatment in NSCLC patients. **(A)** Left panel: TMB in patients with NDB (*n* = 44, median = 6 Mut/Mb) versus patients with DCB (green, *n* = 32, median = 8.5 Mut/Mb) (Mann–Whitney *p* = 0.024). Right panel: TMB in patients with PD (*n* = 38, median = 5 Mut/Mb), SD (blue, *n* = 14, median = 8.5 Mut/Mb), and CR/PR (red, *n* = 24, median = 8.5 Mut/Mb) (Dunn's multiple comparisons test, *p* = 0.018, 0.028, and 0.99). (B) Receiver operating characteristic (ROC) curve to illustrate the ability of TMB to discriminate durable clinical benefit [NDB *n* = 44 versus DCB *n* = 32, AUC 0.65 (95% CI 0.52–0.78), *p* = 0.025]. (C) Percentage of patients with DCB (green) or (D) PD, SD (blue), CR/PR (red) falling into TMB‐low (≤ 4 Mut/Mb), ‐intermediate (5 < *x* < 9 Mut/Mb), and ‐high group (≥ 9 Mut/Mb).

The durable clinical benefit (DCB:CR/PR or SD ≥6 months) rate was 42%. Baseline clinical variables were comparable between patients with TMB‐high and those with TMB‐intermediate/low; however, TMB was significantly higher in patients with DCB than in patients with no benefit (median TMB = 8.5 versus 6.0 Mut/Mb, Mann–Whitney *p* = 0.0244, Figure 2A). Importantly, neither time between biopsy and treatment start, nor treatment line showed any significant association with TMB (supplementary material, Figure S1B,C).

The TMB distribution of patients reaching SD (median TMB = 8.5 Mut/Mb) as best response was significantly different to that of patients with PD (median TMB = 5.0 Mut/Mb), but comparable to the CR/PR group (median TMB = 8.5 Mut/Mb) (Dunn's test *p* = 0.018 and 0.028, respectively) (Figure 2A). Receiver operating characteristic (ROC) showed an AUC of 0.65 (*n* = 76, Figure 2B) and DCB was 64% in TMB‐high patients, as opposed to 33% and 29% (together 31%) in TMB‐intermediate and TMB‐low patients, respectively (Figure 2C). Furthermore, we observed that patients presenting CR/PR or SD as best response together with a high TMB achieved DCB more frequently than patients belonging to the TMB‐intermediate group (Figure 2D).

**Figure 3:**
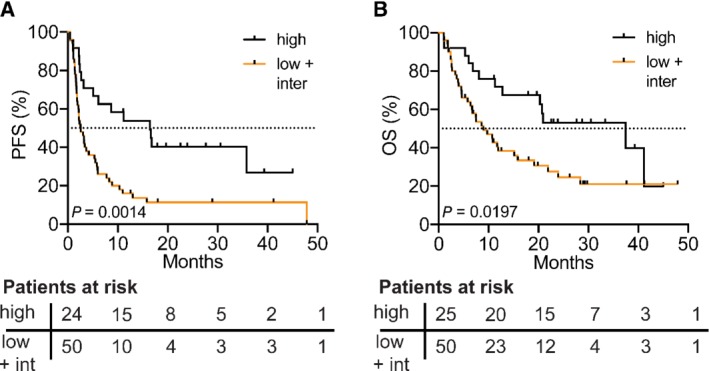
PFS and OS in patients treated with ICI therapy increased in patients with high TMB. (A) PFS from start of immunotherapy in patients with high (≥ 9 Mut/Mb) versus low/intermediate (orange line, < 9 Mut/Mb) TMB [median 16.4 versus 2.6 months, Mantel–Haenszel hazard ratio 0.42 (95% CI 0.25–0.72), log‐rank Mantel–Cox test *p* = 0.0014]. (B) OS from start of immunotherapy in patients with high versus low/intermediate TMB [median 37.5 versus 9.0 months, Mantel–Haenszel hazard ratio 0.51 (95% CI 0.29–0.90), log‐rank Mantel–Cox test, *p* = 0.0197]. Patients at risk according to the time point written on the *x*‐axis of each graph are shown below each plot.

PFS and OS were significantly increased for patients with high TMB when compared with patients with low or intermediate TMB, indicating that TMB is associated with response to ICI treatment (Figure 3A,B). In particular, median PFS was increased from 2.6 months for TMB‐low/intermediate patients to 16.4 months for TMB‐high patients (hazard ratio = 0.42, log‐rank *p* = 0.0014). Similarly, median OS was increased from 9.0 to 37.5 months in TMB‐high patients (hazard ratio = 0.51, log‐rank *p* = 0.0197).

### Gene alterations associated with response to ICI therapy

**Figure 4:**
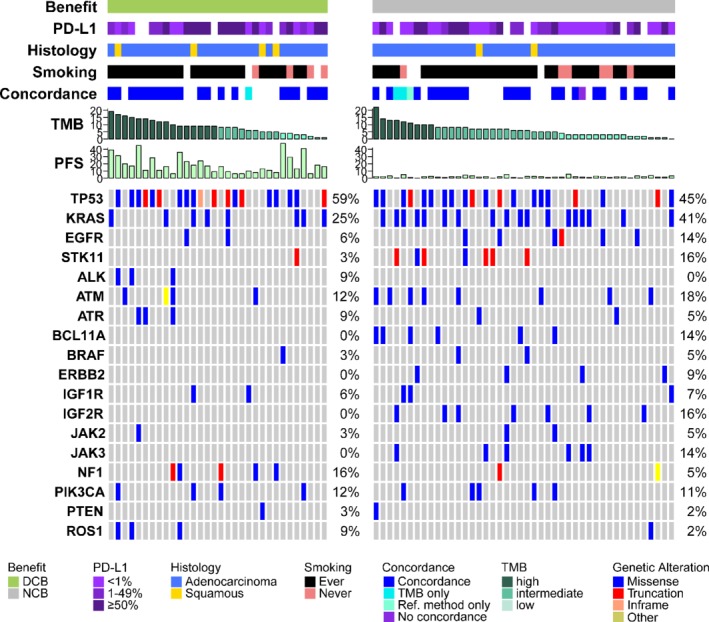
Overview of the clinical and molecular features associated with DCB and NDB in NSCLC patients treated with ICIs. Columns represent individual patients with DCB (green, left panel, *n* = 32) and NCB (grey, right panel, *n* = 44) and are sorted by descending TMB values. PD‐L1 expression is binned into < 1% (light purple), 1–49% (purple), and ≥ 50% (dark purple). Histology distinguishes between adenocarcinoma (blue) and squamous cell carcinoma (yellow). Smoking status is separated into ever‐ (black) and never‐smokers (pink). Concordance indicates the correlation between gene variants detected by the TMB compared with a reference molecular profiling method (further described in the Materials and methods section). TMB is shown in mutations/megabase in descending order and colored according to tertiles (from dark to light green = high to low). PFS is shown in months. Mutation frequencies are shown per gene and variant types are separated into missense (blue), truncation (red), inframe (orange), and other (yellow) variants. Patients for whom clinical data were not available are blank.

Primary and adaptive resistance to immunotherapy has been associated with specific genetic alterations 33, 34, 35, 36. Thus, we sought to assess the genomic features of our patient cohort and their association with response to ICI therapy. First, we compared the variants detected by the TML panel with those identified by the orthogonal sequencing method used at first diagnosis (Materials and methods section). We found an overall concordance of 89%, indicating that the TML panel can also be used for the detection of clinically relevant mutations (Figure 4 and supplementary material, Figure S2A). We identified mutations that were previously associated with resistance to ICIs in our patient cohort, namely *STK11* (seven patients with *STK11* mutations did not respond, whereas one patient showed DCB) (Figure 4). Among all the variants detected in our samples, *IGF2R* and *JAK3* mutations were enriched in the NDB group (*IGF2R* odds ratio 1.38, Fisher's exact *p* = 0.019; *JAK3* odds ratio 1.31, Fisher's exact *p* = 0.036; Figure 4 and supplementary material, Figure S2B) and *MRE11* and *PIK3CG* mutations were enriched in the DCB group (odds ratio 1.28, Fisher's exact *p* = 0.028; Figure 4 and supplementary material, Figure S2B). Consistent with previous reports 15, 37, we found *TP53* mutations to be associated with high TMB, without reaching statistical significance, possibly due to our limited sample size (odds ratio 1.94, Fisher's exact *p* = 0.086; supplementary material, Figure S2C,D). Interestingly, *IGF2R* and *PIK3CG* have been linked to T‐cell regulation and immune response 38, 39. Larger clinical studies focusing on molecular analysis will help to identify recurrent alterations conferring benefit or resistance to ICIs.

### PD‐L1 expression and TML values have complementary predictive power

**Figure 5:**
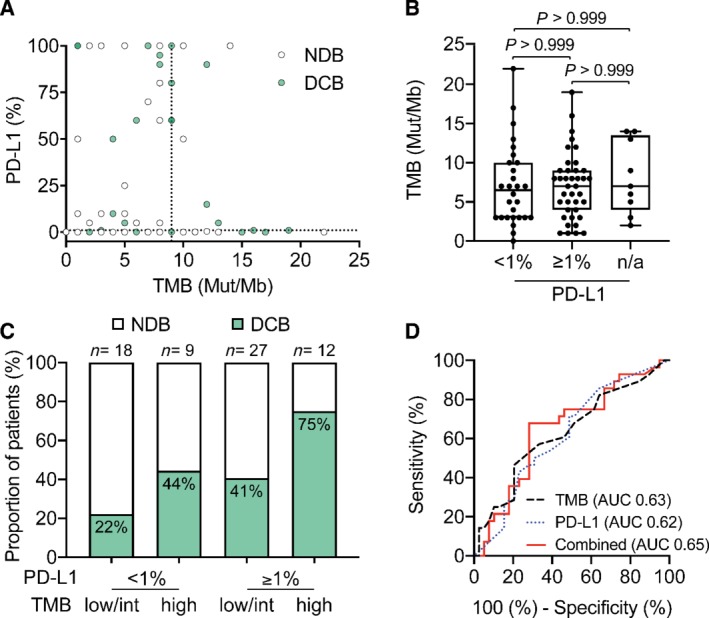
Multivariate analysis of PD‐L1 and TMB improves patient stratification into responders and non‐responders. (A) Correlation between TMB and PD‐L1 expression (*n* = 67, Spearman correlation *r* = 0.003, 95% CI −0.24 to 0.25). The dotted line indicates the cut‐off for TMB‐high classification (9 Mut/Mb). Patients with DCB are colored in green. (B) Distribution of TMB in PD‐L1‐negative (TPS < 1) (*n* = 28, median = 6.5 Mut/Mb), PD‐L1‐positive (TPS ≥1, *n* = 39, median = 7.0 Mut/Mb), and patients with unavailable data (*n* = 9, median = 7.0 Mut/Mb) (Dunn's multiple comparison test, all *P* values > 0.99). (C) Percentage of patients with DCB (green) with status of TMB‐low/int or ‐high in combination with PD‐L1 percentage < 1 or ≥ 1. (D) ROC curves for correlation of TMB (black dashed line, AUC = 0.63) and PD‐L1 expression (blue dotted line) (AUC 0.62) as single biomarkers or combined (red solid line) with DCB (AUC 0.65, 95% CI 0.51–0.78, *p* = 0.0395). Multivariate analysis was calculated using a linear model.

Next, we compared the predictive power of TMB to that of PD‐L1 expression in samples where PD‐L1 staining was available (*n* = 67; supplementary material, Figure S3A). In line with other studies 15, 17, we did not observe a correlation between PD‐L1 expression in tumor cells (TCs) and TMB (Spearman *r* = 0.003; Figure 5A). PD‐L1‐positive (≥ 1%) versus PD‐L1‐negative tumors showed a comparable distribution of TMB values (median 6.5 versus 7.0 Mut/Mb, *p* > 0.999; Figure 5B). However, the highest response rate was observed in PD‐L1‐positive samples with high TMB (75%), whereas the PD‐L1‐negative and TMB‐low/intermediate population only included 22% responders (Figure 5C). Setting the PD‐L1 positivity cut‐off at 50% expressing TCs decreased the sensitivity of identifying responders, consistent with the fact that our cohort mainly comprised patients treated with nivolumab (79%; see ref 40 for a comprehensive review of the recommended PD‐L1 positivity cut‐offs in ICIs and supplementary material, Figure S3B). Lastly, we performed a multivariate ROC analysis, confirming the increased predictive power when both biomarkers are combined (AUC = 0.65 versus 0.63 and 0.62 for TMB and PD‐L1 alone; *n* = 67, respectively; Figure 5D and supplementary material, Figure S3C).

Taken together, our data show the potential of TMB in predicting benefit from ICIs, particularly in combination with additional biomarkers.

## Discussion

In this study, we examined the performance of the Oncomine™ TML assay by retrospectively assessing TMB in tumor tissue specimens from advanced NSCLC patients who had been treated with ICI therapy. We have previously confirmed the analytical validity of the assay, which showed high correlation with tumor–normal‐matched WES data and reproducibility 21. As several commercially available panels for TMB evaluation exist, there is an urgent need for standardization across different assays and diagnostic centers. Multiple harmonization initiatives are ongoing and will provide guidelines for TMB assessment. The Oncomine™ TML assay is currently being evaluated as part of one of these initiatives, which directly analyzes the assay reproducibility across multiple centers and compares its performance with FDA‐approved diagnostic tests (personal communication with Thermo Fisher European Immuno‐Oncology Consortium).

Our sample cohort consisted of tissue specimens previously profiled as part of standard clinical care. This is the first report demonstrating the clinical validity of the Oncomine™ TML assay in predicting NSCLC patient response to ICI treatment in a routine clinical laboratory. Our data show that high TMB is significantly associated with durable clinical benefit from ICI therapy. Patients with TMB ≥ 9 mutations (Mut)/Mb have an increased likelihood of benefiting from ICIs, made evident by increased PFS as well as increased OS compared with patients with TMB < 9 Mut/Mb. Previous studies have shown that TMB is not a positive prognostic factor *per se* 17, 41; thus, it is reasonable to assume that the association of TMB with increased survival in this study can be directly linked to response to ICI treatment.

Importantly, our patient cohort consisted of a very heterogeneous population. The number of treatment lines prior to ICI treatment as well as the time from biopsy acquisition to ICI treatment was highly variable. However, despite this heterogeneity, TMB association with treatment response was significant, underlining the robustness and potential power of TMB as a predictive biomarker. We did observe a significant difference in TMB between metastatic and non‐metastatic tumors (Table 1). This is likely due to the accumulation of mutations during the evolution of a tumor, a phenomenon that has been described before 42.

To assess TMB routinely in a clinical laboratory and use it for clinical decision‐making, it is important to address key pre‐analytical factors. First, we evaluated the impact of synonymous mutations on TMB estimation. From a biological point of view, synonymous mutations are unlikely to contribute to immunogenicity. However, they may help to increase precision when using targeted panels by increasing the total number of mutations used for TMB calculation 20, 22. We evaluated this hypothesis by comparing the TMB values obtained using either only non‐synonymous mutations or all mutations but observed a high correlation between the two TMB datasets (Figure 1C). Nevertheless, it is an important point to consider when comparing commercially available assays, as it will directly affect the TMB cut‐off used for patient stratification.

Second, formalin fixation of tissue specimens, a required process for the generation of FFPE blocks, may result in deamination of cytosine nucleotides, ultimately leading to false‐positive C:G>T:A mutations 29. While this is a general issue when performing DNA analyses on FFPE material, it is particularly problematic when assessing TMB. Even a few false‐positive variants may strongly affect the TMB value and potentially lead to wrong classification of patient samples. We demonstrated that treating DNA samples with UDG prior to library preparation significantly reduces the presence of potential deamination artifacts (Figure 1B). We therefore recommend routinely treating DNA with UDG when assessing TMB.

Finally, here we define the cut‐off for high mutational load by using the upper tertile of the TMB distribution, an approach that other studies have also used 14, 32. Alternatively, some studies have defined the cut‐off for high mutational burden as the median TMB value 15, 16. Using this last approach (cut‐off at a median of 7 Mut/Mb; supplementary material, Figure S4), we still observe a significant correlation between high TMB and DCB as well as PFS (supplementary material, Figure S4A–C). However, OS is no longer significantly increased, indicating that using the upper TMB tertile as a cut‐off for stratification might be a better predictor of long‐term clinical benefit (supplementary material, Figure S4D,E).

Despite the high durable clinical benefit rate (64%) in the top TMB tertile, there is a fraction of patients with a high mutational load (≥ 9 Mut/Mb) but no DCB. While some of these cases may also be related to the heterogeneity of our sample cohort, several studies have suggested different mechanisms that may confer resistance to ICI treatment independent of TMB status 33, 34, 35, 36, 43. These include alterations to signaling pathways such as the MAPK, PI3K, IFN, and WNT pathways (reviewed in ref 33). To investigate this in our cohort, we evaluated the mutational profiles of all tested samples. Although the statistical power of this analysis is limited by sample size, we do observe variants in genes that have been linked to ICI resistance. We detected mutations significantly enriched in the NDB group (*IGF2R* and *JAK3* mutations) and in the DCB group (*MRE11*and *PIK3CG* mutations) (supplementary material, Figure S2B). Furthermore, we identified seven patients presenting *STK11* mutations (five of which together with *KRAS* mutations) in the high and intermediate TMB group who did not respond to therapy (Figure 4). Together, these data confirm previous reports suggesting that specific mutations may influence the likelihood of responding to ICIs.

Moreover, we evaluated how TMB compares to PD‐L1 expression as a predictive biomarker. In line with previous reports, we observed no direct correlation between the two markers, yet the predictive power of each biomarker alone was comparable. However, performing a multivariate analysis with the two markers yielded increased performance for predicting therapy response (Figure 5D), confirming other reports that suggest a combinatorial approach for stratifying patients for ICI therapy 14, 15, 17.

Lastly, while commercial tests performed by centralized laboratories offer TMB analysis as part of their routine molecular tests, there are clear advantages of analyzing TMB locally. First, when run in‐house, the test can be performed significantly cheaper, resulting in reduced healthcare costs and making it more accessible to patients. Second, the quality of molecular tumor boards is highly increased when molecular profiles including TMB can be discussed directly with the experts who have conducted the tests. Third, a well‐organized in‐house laboratory setup may have a significantly lower TaT for testing TMB than a centralized laboratory, increasing the quality of care for the patient.

Taken together, our study clearly demonstrates the clinical validity of using TMB as a predictive biomarker for ICI therapy. However, we also show that integration of different biomarkers may be the most predictive approach for clinical decision‐making for ICI therapy. Therefore, the identification and integration of further biomarkers such as PD‐1 expression in T cells 44, T‐cell receptor repertoire 45, 46, 47, and gene expression profiling of the tumor microenvironment 48 (reviewed in 49, 50) will be key to further increasing the predictive power of multivariate molecular profiling.

## Author contributions statement

PJ and LQ conceived the idea for the study. PJ supervised the study. IA, KL, SIR, and PJ interpreted the data and wrote the manuscript. IA, PJ, and LQ planned the experiments. IA, KL, LPL, and JH performed and analyzed the experiments. SIR, SP, KDM, and MB collected and analyzed the clinical data. IA, KL, LPL, and PJ performed the bioinformatics analysis of the sequencing data. MT, AZ, and HL provided administrative and material support. SSP, LB, and KDM analyzed and interpreted the PD‐L1 data. SSP and KDM performed histological analyses.

## Supporting information


**Supplementary Figure Legends**

**Figure S1.** Pre‐analytical factors affecting TMB measurements
**Figure S2.** (Extends over six image files.) Full list of detected variants and concordance between TML panel and reference NGS method in NSCLC patients treated with ICIs
**Figure S3.** PD‐L1 cut‐off at 50% is less predictive than that at 1%
**Figure S4.** Cut‐off at median shows no significant gain in OS
