# Supplementary material for: Tumor mutational burden assessed by targeted NGS predicts clinical benefit from immune checkpoint inhibitors in non‐small cell lung cancer
Source: J Pathol. 2019 Oct 24;250(1):19–29. doi: 10.1002/path.5344 (PMC6972587; doi:10.1002/path.5344)
Supplement: Supplementary file 1 — Supplementary Figure Legends [file PATH-250-19-s001.docx]

**Tumor mutational burden assessed by targeted NGS predicts clinical benefit from immune checkpoint inhibitors in non-small cell lung cancer**

Alborelli I *et al. J Pathol* DOI: 10.1002/path.5344

**Supplementary figure legends**

**Figure S1. Pre-analytical factors affecting TMB measurements.**

(A) Distribution of TMB values including synonymous mutations across the sample cohort (*n* = 76). Solid line indicates the median of all samples (8.5 Mut/Mb). (B) Distribution of the time between biopsy to treatment start across all samples, samples where TMB predicted therapy outcome (concordant), and samples where TMB prediction does not match the clinical outcome. (C) Distribution of TMB values across samples sorted by the line of treatment.

**Figure S2. (Extends over six image files.) Full list of detected variants and concordance between TML panel and reference NGS method in NSCLC patients treated with ICIs.**

(A) Representation of the concordance between variants detected by the TML panel and a reference molecular profiling method. Perfect concordance was observed for 89% of the samples, whereas the TML panel detected additional variants in 6% of the samples and the reference method detected additional variants in 2% of the samples. No concordance was observed for one sample (2%). Only target regions covered by both TML and reference method were considered for the analysis. (B, C) Analysis of the enrichment for specific gene alterations in the durable clinical benefit (DCB) versus no durable benefit (NDB) (B) and tumor mutational burden (TMB) high (C). Log_2_[odds ratio (OR)] and –log_2_(*P* value) are represented on the *X-* and *Y*-axis, respectively. Dashed lines indicate cut-off for significance (*Y*-axis, *p* = 0.05) and OR = 0 (*X*-axis). Only the 24 most significant genes from our analysis are depicted. (D) Columns represent individual patients with DCB (green, left panel, *n* = 32) and NCB (grey, right panel, *n* = 44) and sorted by descending TMB values. PD-L1 expression is binned into < 1% (light purple), 1–49% (purple), ≥ 50% (dark purple). Histology distinguishes between adenocarcinoma (blue) and squamous cell carcinoma (yellow). Smoking status is separated into ever- (black) and never-smokers (pink). Concordance indicates the correlation between gene variants detected by the TMB compared with a reference NGS panel (further described in the Materials and methods section). TMB is shown in mutations/megabase in descending order and colored according to tertiles (from dark to light green = high to low). PFS is shown in months. Mutation frequencies are shown per gene and variant types are separated into missense (blue), truncation (red), inframe (orange), and other (yellow) variants. Patients for whom clinical data were not available are blank.

**Figure S3. PD-L1 cut-off at 50% is less predictive than that at 1%.**

(A) Distribution of TMB values with availability of PD-L1 expression data shown in red. (B) Percentage of patients with DCB (green) with TMB low/intermediate or high in combination with PD-L1 percentage < 50 or ≥ 50%. (C) Comparison of specificity, sensitivity, and positive predictive value (%) (NDB, *n* *=* 39 versus DCB, *n* *=* 28, total *n* *=* 67).

**Figure S4. Cut-off at median shows no significant gain in OS.**

(A) Percentage of patients with DCB (green) or (B) PD, SD (blue), CR/PR (red) falling into TMB-low (< 7 Mut/Mb), or -high group (≥ 7 Mut/Mb) according to median cut-off. (C) PFS from start of immunotherapy in patients with high (≥ 7 Mut/Mb) versus low (orange line, < 7 Mut/Mb) TMB (median 6.0 versus 2.6 months, Mantel–Haenszel hazard ratio 0.59, 95% CI 0.35–0.996, log-rank Mantel–Cox test *p* = 0.0482). (D) OS from start of immunotherapy in patients with high versus low/intermediate TMB (median 15.1 versus 10.7 months, Mantel–Haenszel hazard ratio 0.93, 95% CI 0.53–1.64, log-rank Mantel–Cox test *p* = 0.802). (E) Tabular comparison of median month progression-free and overall survival in all the different TMB subgroups defined.
