# Supplementary figures and images for: Tumor mutational burden assessed by targeted NGS predicts clinical benefit from immune checkpoint inhibitors in non‐small cell lung cancer
Source: J Pathol. 2019 Oct 24;250(1):19–29. doi: 10.1002/path.5344 (PMC6972587; doi:10.1002/path.5344)

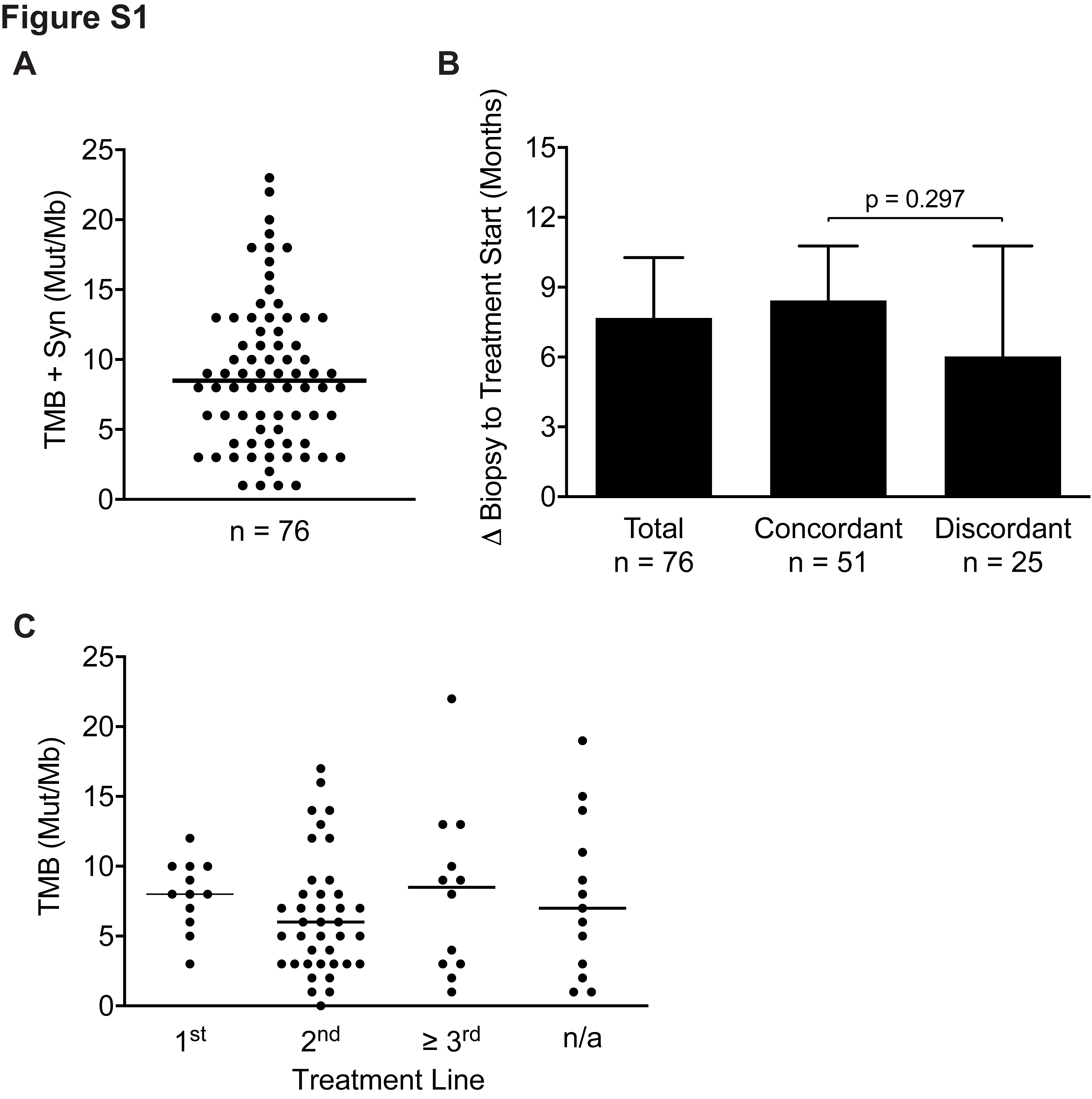

Supplement: Supplementary file 2 — Figure S1. Pre‐analytical factors affecting TMB measurements Figure S2. (Extends over six image files.) Full list of detected variants and concordance between TML panel and reference NGS method in NSCLC patients treated with ICIs Figure S3. PD‐L1 cut‐off at 50% is less predictive than that at 1% Figure S4. Cut‐off at median shows no significant gain in OS [file PATH-250-19-s002.zip › path_5344_Supp_Figure_S1.tif]

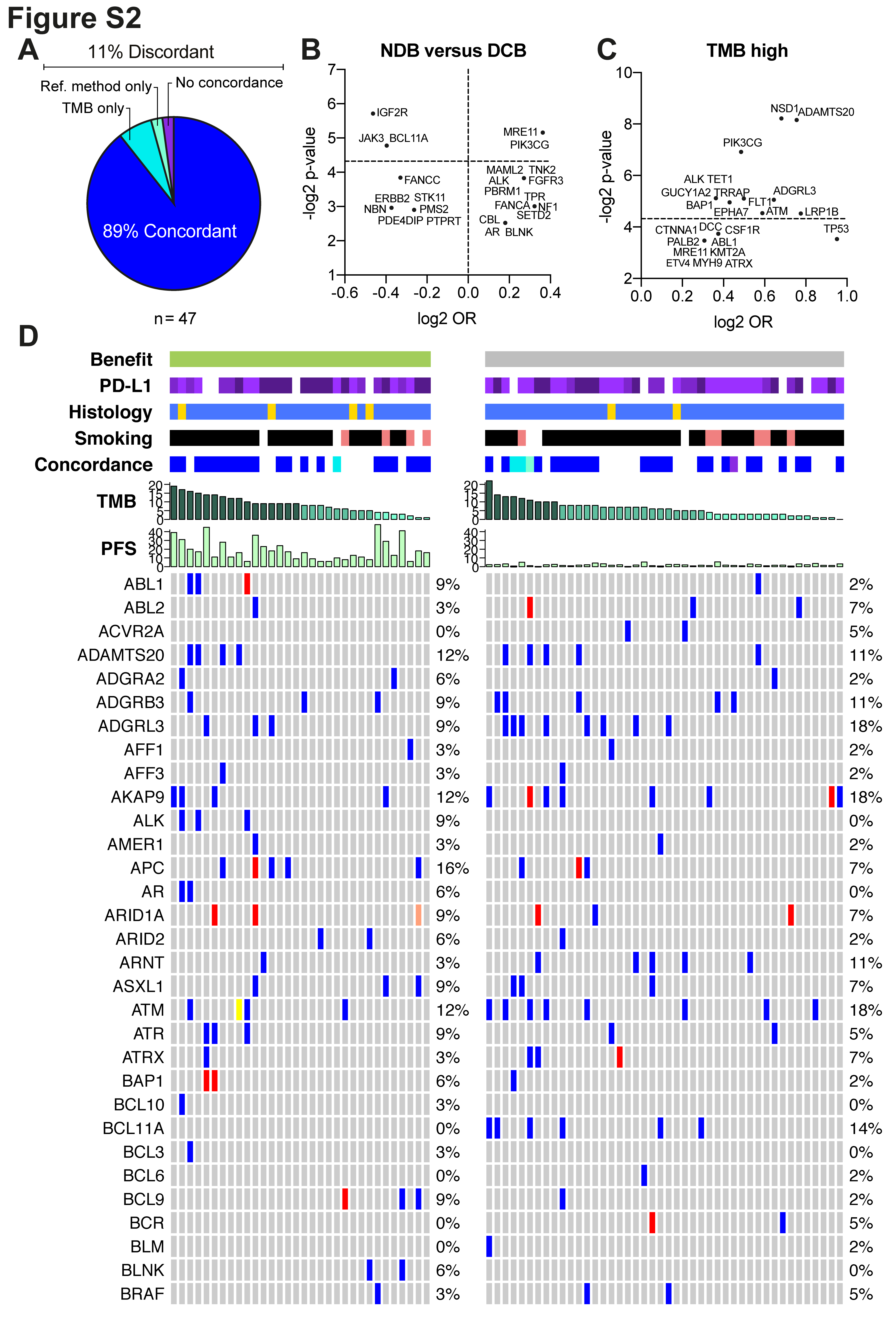

Supplement: Supplementary file 2 — Figure S1. Pre‐analytical factors affecting TMB measurements Figure S2. (Extends over six image files.) Full list of detected variants and concordance between TML panel and reference NGS method in NSCLC patients treated with ICIs Figure S3. PD‐L1 cut‐off at 50% is less predictive than that at 1% Figure S4. Cut‐off at median shows no significant gain in OS [file PATH-250-19-s002.zip › path_5344_Supp_Figure_S2.1-01.tif]

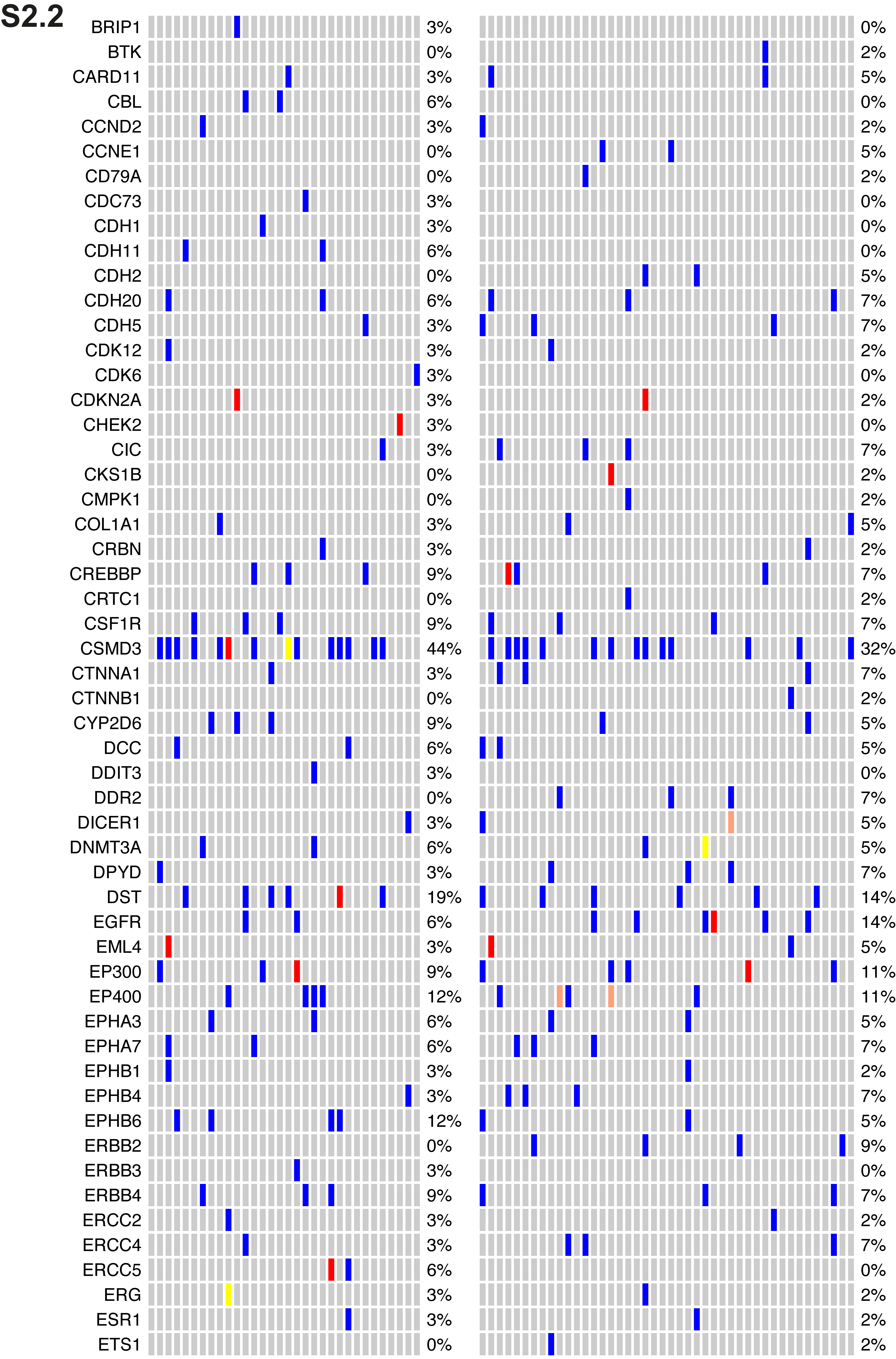

Supplement: Supplementary file 2 — Figure S1. Pre‐analytical factors affecting TMB measurements Figure S2. (Extends over six image files.) Full list of detected variants and concordance between TML panel and reference NGS method in NSCLC patients treated with ICIs Figure S3. PD‐L1 cut‐off at 50% is less predictive than that at 1% Figure S4. Cut‐off at median shows no significant gain in OS [file PATH-250-19-s002.zip › path_5344_Supp_Figure_S2.2.tif]

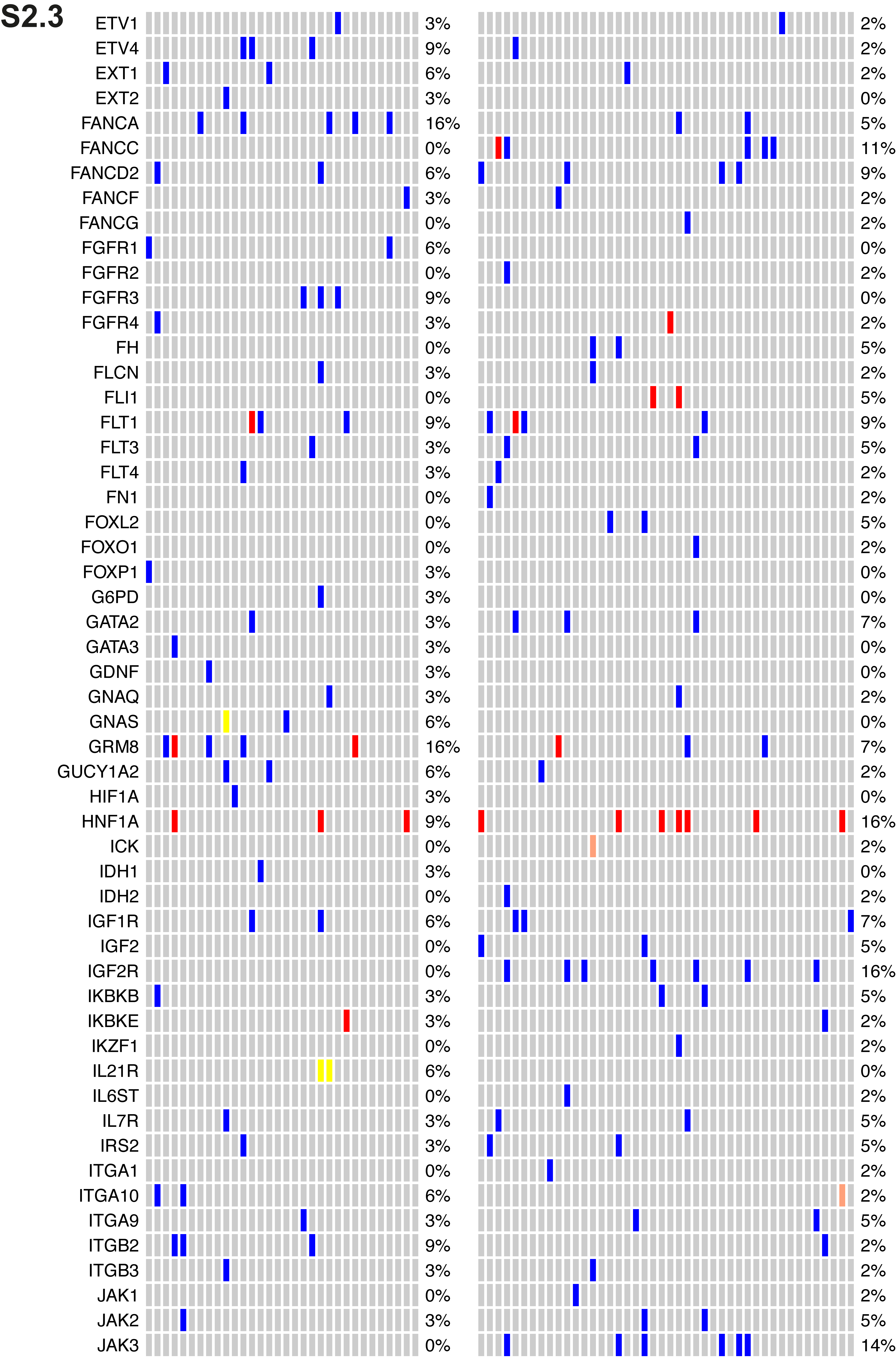

Supplement: Supplementary file 2 — Figure S1. Pre‐analytical factors affecting TMB measurements Figure S2. (Extends over six image files.) Full list of detected variants and concordance between TML panel and reference NGS method in NSCLC patients treated with ICIs Figure S3. PD‐L1 cut‐off at 50% is less predictive than that at 1% Figure S4. Cut‐off at median shows no significant gain in OS [file PATH-250-19-s002.zip › path_5344_Supp_Figure_S2.3.tif]

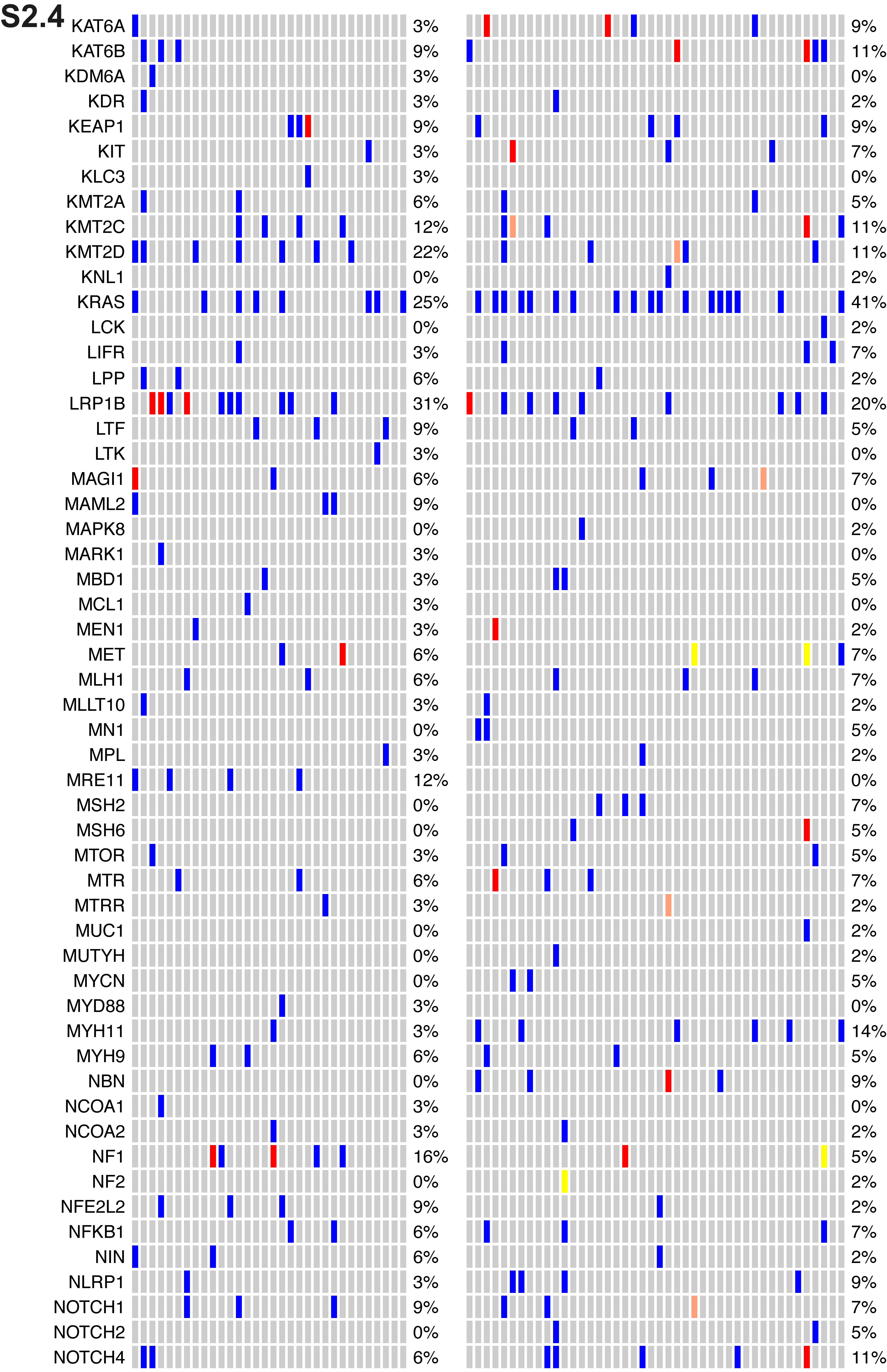

Supplement: Supplementary file 2 — Figure S1. Pre‐analytical factors affecting TMB measurements Figure S2. (Extends over six image files.) Full list of detected variants and concordance between TML panel and reference NGS method in NSCLC patients treated with ICIs Figure S3. PD‐L1 cut‐off at 50% is less predictive than that at 1% Figure S4. Cut‐off at median shows no significant gain in OS [file PATH-250-19-s002.zip › path_5344_Supp_Figure_S2.4.tif]

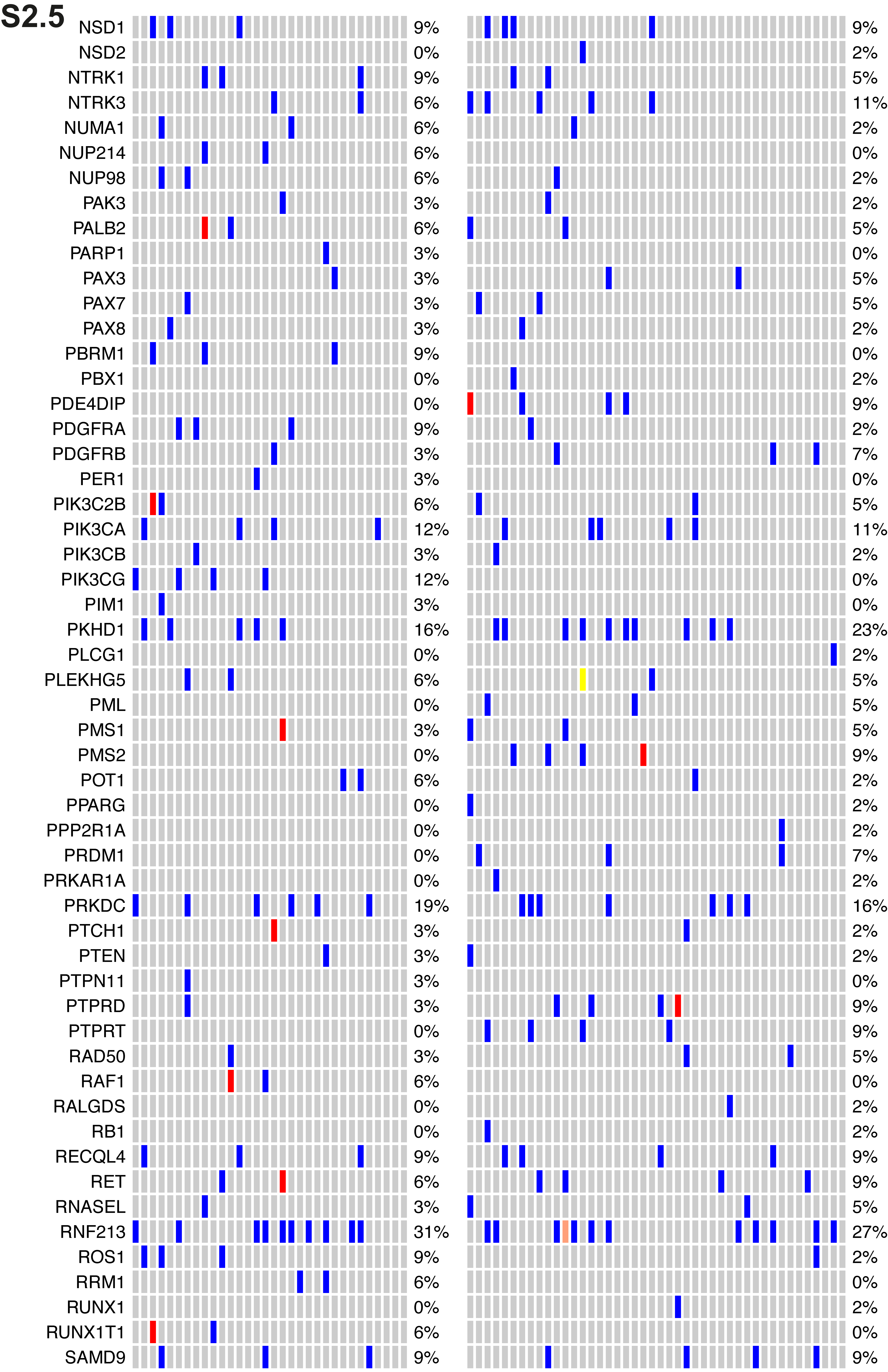

Supplement: Supplementary file 2 — Figure S1. Pre‐analytical factors affecting TMB measurements Figure S2. (Extends over six image files.) Full list of detected variants and concordance between TML panel and reference NGS method in NSCLC patients treated with ICIs Figure S3. PD‐L1 cut‐off at 50% is less predictive than that at 1% Figure S4. Cut‐off at median shows no significant gain in OS [file PATH-250-19-s002.zip › path_5344_Supp_Figure_S2.5.tif]

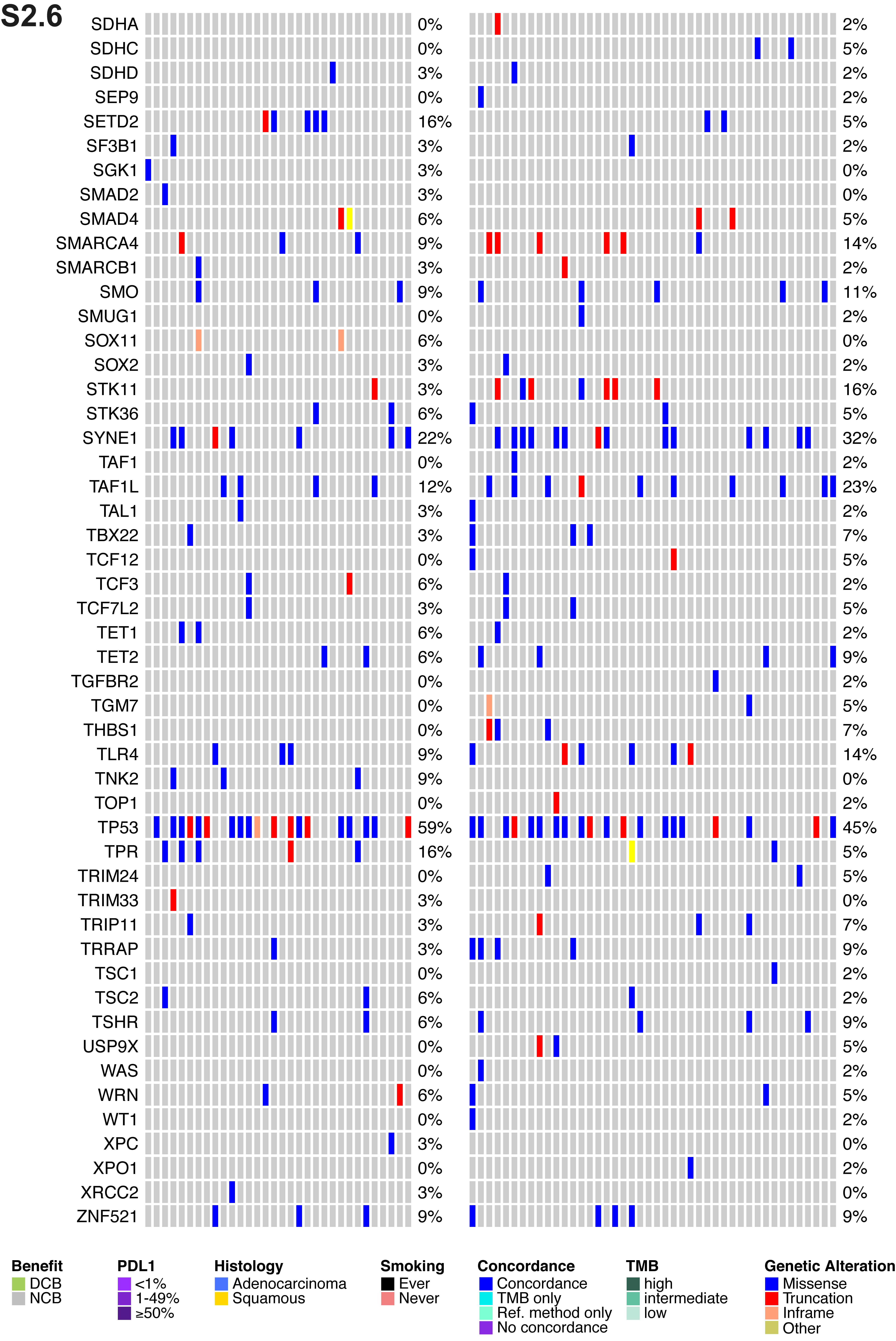

Supplement: Supplementary file 2 — Figure S1. Pre‐analytical factors affecting TMB measurements Figure S2. (Extends over six image files.) Full list of detected variants and concordance between TML panel and reference NGS method in NSCLC patients treated with ICIs Figure S3. PD‐L1 cut‐off at 50% is less predictive than that at 1% Figure S4. Cut‐off at median shows no significant gain in OS [file PATH-250-19-s002.zip › path_5344_Supp_Figure_S2.6.tif]

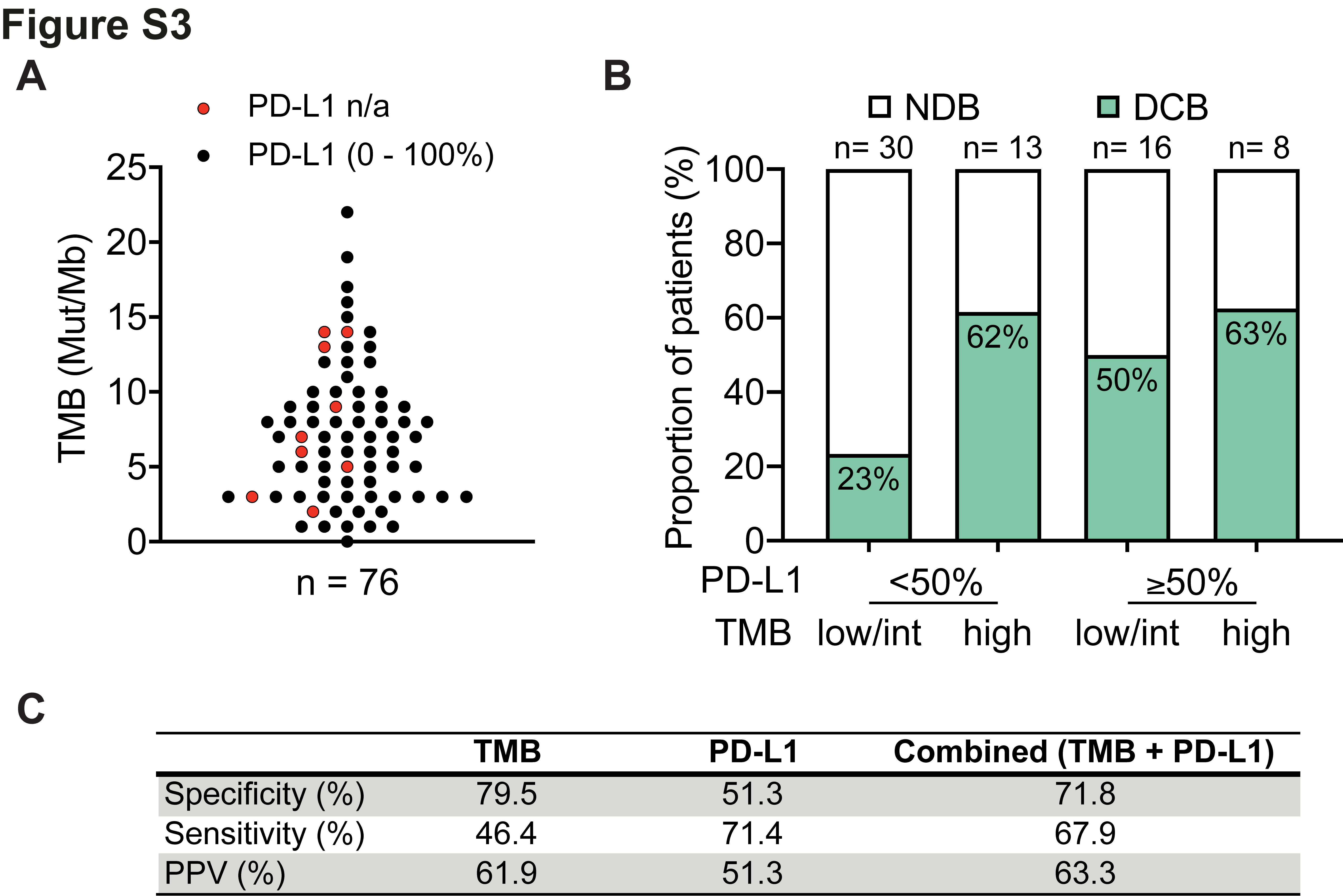

Supplement: Supplementary file 2 — Figure S1. Pre‐analytical factors affecting TMB measurements Figure S2. (Extends over six image files.) Full list of detected variants and concordance between TML panel and reference NGS method in NSCLC patients treated with ICIs Figure S3. PD‐L1 cut‐off at 50% is less predictive than that at 1% Figure S4. Cut‐off at median shows no significant gain in OS [file PATH-250-19-s002.zip › path_5344_Supp_Figure_S3.tif]

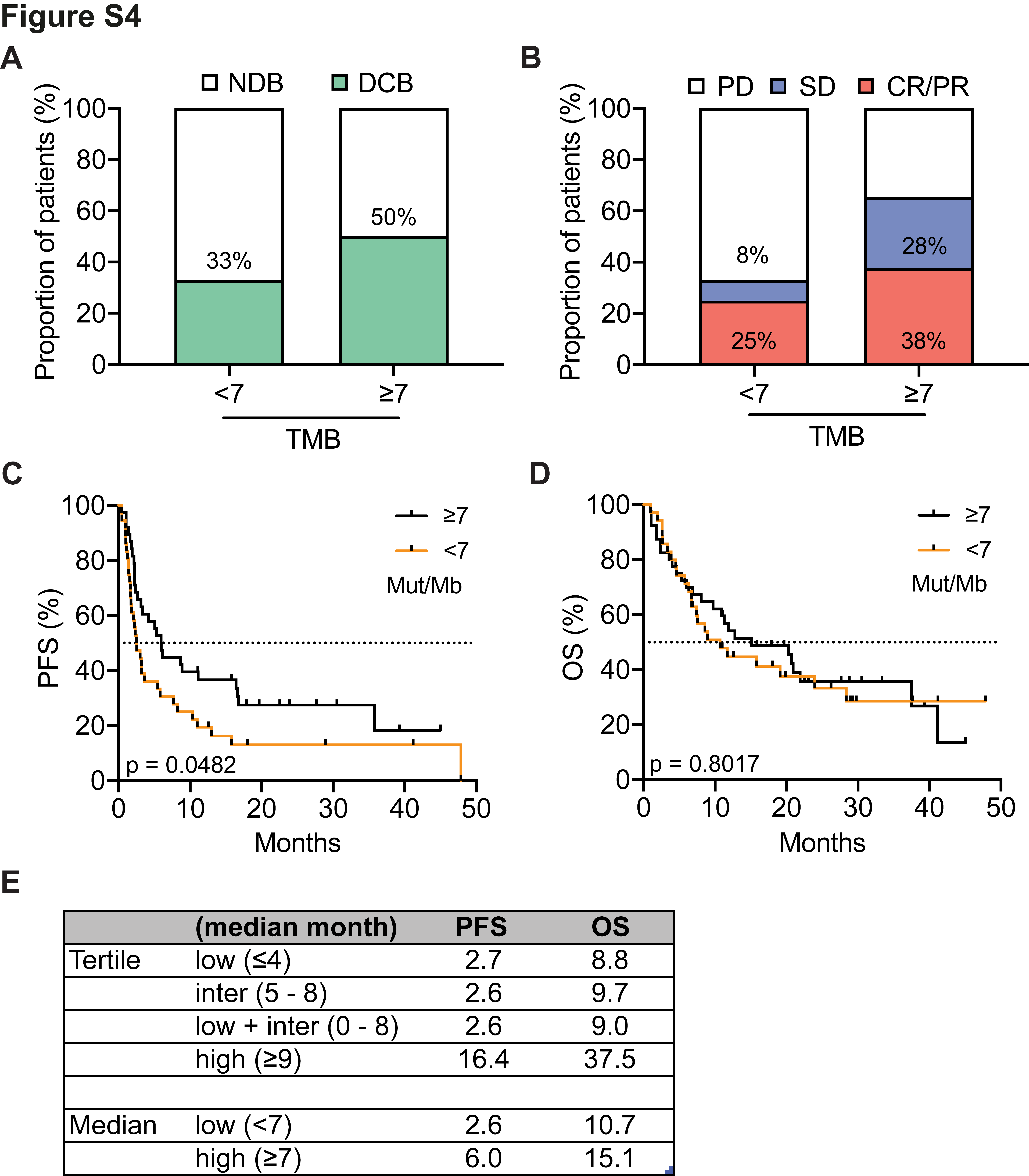

Supplement: Supplementary file 2 — Figure S1. Pre‐analytical factors affecting TMB measurements Figure S2. (Extends over six image files.) Full list of detected variants and concordance between TML panel and reference NGS method in NSCLC patients treated with ICIs Figure S3. PD‐L1 cut‐off at 50% is less predictive than that at 1% Figure S4. Cut‐off at median shows no significant gain in OS [file PATH-250-19-s002.zip › path_5344_Supp_Figure_S4.tif]
